# Center of pressure (COP) measurement in patients with confirmed successful outcomes following shoulder surgery show significant sensorimotor deficits

**DOI:** 10.1007/s00167-021-06751-0

**Published:** 2021-11-06

**Authors:** Yannick J. Ehmann, Daniel P. Berthold, Sven Reuter, Knut Beitzel, Robin Köhler, Fabian Stöcker, Lukas N. Muench, Jonas Pogorzelski, Marco-Christopher Rupp, Sepp Braun, Andreas B. Imhoff, Stefan Buchmann

**Affiliations:** 1 Department of Orthopedic Sports Medicine, Technical University of Munich, Ismaninger Str. 22, 81675 Munich, Germany; 2 SRH University for Applied Health Sciences, Stuttgart, Germany; 3 Atos Orthoparc Klinik, Cologne, Germany; 4 Department of Sport and Health Sciences, TU Munich, Munich, Germany; 5 Gelenkpunkt, Sport and Joint Surgery Innsbruck, Innsbruck, Austria; 6 OSMI - Research Unit for Orthopaedic Sports Medicine and Injury Prevention, UMIT - Private University for Health Sciences, Medical Informatics and Technology, Hall, Austria; 7 OFZ Weilheim, Weilheim, Germany

**Keywords:** Centre of pressure, COP, Shoulder injury, Rehabilitation of shoulder, Sensorimotor function, Rehabilitation

## Abstract

**Purpose:**

To determine the sensorimotor and clinical function of patients with confirmed successful outcome after either undergoing acromioclavicular joint (ACJ) stabilization, Bankart repair (BR), or rotator cuff repair (RC), and to compare these measures to the contralateral, healthy side without history of previous injuries or surgeries of the upper extremity. It was hypothesized that patients of each interventional group would have inferior sensorimotor function of the shoulder joint compared to the contralateral, healthy side, while presenting with successful clinical and functional outcomes.

**Methods:**

Three intervention groups including ten patients who had confirmed successful clinical and functional outcomes after either undergoing ACJ stabilization, BR, or RC were evaluated postoperatively at an average follow-up of 31.7 ± 11.6 months. Additionally, a healthy control group (CG) of ten patients was included. Clinical outcomes were assessed using the Constant–Murley (CM) and American Shoulder and Elbow Surgeons (ASES) Score. Pain was evaluated using the visual analogue scale (VAS). Sensorimotor function was assessed by determining the center of pressure (COP) of the shoulder joint in a one-handed support task in supine position on a validated pressure plate.

**Results:**

Each interventional group demonstrated excellent clinical outcome scores including the CM Score (ACJ 83.3 ± 11.8; BR 89.0 ± 10.3; RC 81.4 ± 8.8), ASES Score (ACJ 95.5 ± 7.0; BR 92.5 ± 9.6; RC 96.5 ± 5.2), and VAS (ACJ 0.5 ± 0.9; BR 0.5 ± 0.8; RC 0.5 ± 0.8). Overall, the CG showed no significant side-to-side difference in COP, whereas the ACJ-group and the BR-group demonstrated significantly increased COP compared to the healthy side (ACJ 103 cm vs. 98 cm, *p* = 0.049; BR: 116 cm vs. 102 cm, *p* = 0.006). The RC-group revealed no significant side-to-side difference (120 cm vs. 108 cm, n.s.).

**Conclusion:**

Centre of pressure measurement detected sensorimotor functional deficits following surgical treatment of the shoulder joint in patients with confirmed successful clinical and functional outcomes. This may indicate that specific postoperative training and rehabilitation protocols should be established for patients who underwent surgery of the upper extremity. These results underline that sensorimotor training should be an important component of postoperative rehabilitation and physiotherapeutic activities to improve postoperative function and joint control.

**Level of evidence:**

IV.

## Introduction

Postoperative limited sensorimotor function compromises sufficient joint stability along with an increased risk of future re-injuries [1–3]. The sensorimotor system has been reported to play an integrative role by mediating static and dynamic stabilizers, ensuring optimal function and stability of the shoulder joint [1, 4–7].

Recently, Edouard et al. implemented a novel testing protocol to assess the sensorimotor function of the shoulder [2]. Deficits of the afferent, central and efferent sensorimotor system, strength, or coactivation have been highlighted in patients with glenohumeral joint instability by some variations of COP [1, 2, 8, 9]. Unfortunately, detailed knowledge regarding sensorimotor shoulder function following surgery and their impact on clinical outcomes remains limited. However, this is of clinical relevance, as improved sensorimotor function may lead to better clinical outcomes, higher patient satisfaction, and reduced risk of re-injury in the long term.

The purpose of the study was to determine the sensorimotor and clinical function of patients with confirmed successful outcome, and to compare these measures to patients without history of previous injuries or surgeries of the upper extremity. It was hypothesized that patients of each interventional group would have inferior sensorimotor function of the shoulder joint compared to the contralateral side, while presenting with successful clinical and functional outcomes.

## Materials and methods

Ethical approval was obtained via Human Research Determination Form to the institutional review board (IRB) of the Technical University of Munich (IRB #64/14). A retrospective chart review was performed on patients undergoing surgery of the upper limb between 01/2012 and 06/2015 at the author’s institution. Patients were eligible for inclusion if they underwent either acute (primary) ACJ reconstruction after sustaining a Rockwood IV or V injury [10, 11], primary rotator cuff repair, or primary Bankart repair after suffering from anterior instability of the glenohumeral joint without a significant bone loss (defined as < 15%) [12], and had confirmed successful postoperative outcomes, defined as the absence of restrictions in clinical outcome scores and pain. Patients were excluded if they had injuries of the contralateral shoulder, neurovascular injuries, fractures, concomitant injuries to the upper limb other than ACJ instabilities (Rockwood IV, V), reconstructable isolated supraspinatus rotator cuff tears, or Bankart lesions or if they had any kind of restricting pain in their shoulders, wrists, elbows, or upper back. The first ten consecutive patients of each group to fulfill these criteria were included. Additionally, ten healthy patients without prior injuries or surgeries of the upper limb were included to serve as a control group. Minimum follow-up was 12 months.

Based on the type of injury, patients were allocated to groups: (1) arthroscopic ACJ reconstruction using a Tightrope and Endobutton (Arthrex Inc., Naples, FL, USA) repair after isolated acute ACJ injuries Rockwood type IV-V (ACJ) [13], (2) rotator cuff repair using a double-row Speedbridge configuration (Arthrex Inc., Naples, FL, USA) (RC) [14], (3) arthroscopic Bankart repair after suffering from anterior instability (BR) [15], and (4) healthy control group (CG).

### Clinical outcome scores

The Constant–Murley (CM) Score was used to evaluate the level of pain and the ability to carry out normal daily activities. The American Shoulder and Elbow Surgeons (ASES) Score was used to measure shoulder pain and functional limitations in patients with musculoskeletal complaints. Pain was measured using a visual analogue scale (VAS). Previous studies have confirmed these scores in terms of reliability, validity, and responsiveness.

### Postoperative rehabilitation

Each patient underwent a structured postoperative rehabilitation protocol for the first 3 months postoperatively, while shoulder braces were recommended for 6 weeks. Rehabilitation began on the first postoperative day with range of motion (ROM) being limited after rotator cuff repair as follows: weeks 1–3: passive flexion, abduction 90/0/0°; weeks 4–6: active flexion, abduction 90/0/0°. ROM after Bankart Repair was limited to: weeks 1–3: active flexion, abduction 45/0/0°; active internal/external rotation 80/0/0°; weeks 4–6: active flexion and abduction to 90/0/0°; active internal/external rotation to 80/0/0°. Finally, ROM after ACJ-repair was limited: weeks 1–2: active flexion, abduction 30/0/0°; active internal/external rotation 80/0/15°; weeks 3–4: active flexion, abduction 45/0/0°; active internal/external rotation 80/0/30°; weeks 4–6: flexion, active abduction 90/0/0° [16]. For all groups, no specific sensorimotor training was performed.

### Sensorimotor function testing

The analysis of sensorimotor abilities was conducted using a static force platform (Bertec Corporation, Columbus, OH, USA). This strain-gauge-based technology detects vertical and horizontal forces, and torques which enables calculating the sway of the center of pressure (COP) for the shoulder joint [2, 8], sampled at 100 Hz.

Testing was conducted in a standardized setting described by Edouard et al. [2, 8]. The subjects were in a one-handed prone position with the lower part of the body supported on an adjustable height table up to the anterior superior iliac spines with the hands on the force platform, while the other hand was positioned on the belly. The height of the table was adjusted to allow the upper limbs to remain outstretched in 90° of shoulder flexion with the hands placed on the platform. The elbows were in full extension, and the wrists were at 90° extension to place the upper limbs at 90° to the platform and the ground. The tests were performed in a noise-free environment, with no variation in luminosity. To avoid inter-tester variability, all evaluations were supervised by the same person.

A familiarization period supported on both hands with eyes open preceded each test. Tests were performed in four conditions, always in the same order: eyes open supported on the dominant side (EO-DS), eyes closed supported on the dominant side (EC-DS), eyes open supported on the non-dominant side (EO-NS), and eyes closed supported on the non-dominant side (EC-NS). The recorded tests started 5 s after the subject maintained the test position and lasted for 25 s. A 30 s period rest was given between each condition. The measurement accuracy was full centimeters.

### Statistical analysis

All variables were evaluated for distribution of normality using a combination of histograms, quantile–quantile (Q–Q) plots, and Shapiro–Wilk tests. Descriptive statistics were summarized as means and standard deviations for quantitative variables and as counts and frequencies for categorical variables. The COP was summarized as medians, 25% quartiles, and 75% quartiles. The significance of mean differences between continuous, normally distributed data was evaluated using paired- and independent-samples t tests. The significance of mean differences between continuous, non-normally distributed data was evaluated using non-parametric h tests (Kruskal and Wallis). The incidence between groups was assessed using Chi-square or Fisher’s exact tests. Statistical significance for all comparisons was set at *P* < 0.05. All analyses were performed with Stata statistical software (StataCorp. 2017. Stata Statistical Software: Release 15. College Station, TX: StataCorp LLC). A post hoc power analysis was conducted for comparison of the center of pressure measurement using a two-sided test. With an *α* of 0.05, it was shown that the sample size in this study could achieve an adequate power of 0.92. The sample size calculation and the power analysis were performed using G*power 3.1.

## Results

The first ten consecutive patients to meet the inclusion and exclusion criteria in each group were included. As such, the final study cohort comprised 40 patients with 10 patients in each respective group. The average time between surgery and final testing in the intervention groups was 31.7 ± 11.6 months. Additional demographic data for the groups and the statistical analysis are delineated in Table 1.

**Table 1:** Demographic data of the patient cohort

	Gender (*n*)		Age surgery (y)		Age test (y)		Time between surgery and test (mo)		Affected side (*n*)	
	m	f	Mean	SD	Mean	SD	Mean	SD	Dominant	Non-dominant
ACJ-group	9	1	35.4	12.5	38.1	12.7	29.6	5.3	7	3
RC-group	5	5	58.8	7.3	61.4	7.8	35.1	14.9	7	3
BR-group	9	1	29.4	8.7	31.9	9.2	30.4	12.6	4	6
Control-group	7	3	–	–	47.0	21.0	–	–	–	–

*n* number, *m* male, *f* female, *SD* standard deviation, *y* years, *mo* months, *ACJ-group* acromioclavicular joint group, *RC-group* rotator cuff repair group, *BR group* Bankart repair group

### Clinical outcome scores

Overall, each interventional group showed excellent postoperative clinical outcome scores at a minimum follow-up of 12 months. Clinical outcomes are delineated in Table 2.

**Table 2:** Clinical outcome data of the patient cohort

	CM		ASES		VAS	
	Mean	SD	Mean	SD	Mean	SD
ACJ-group	83.3	11.8	95.5	7	0.5	0.9
RC-group	81.4	8.8	92.5	9.6	0.5	0.8
BR-group	89	10.3	96.5	5.2	0.5	0.8

*ACJ-group* acromioclavicular joint group, *RC-group* rotator cuff repair group, *BR-group* Bankart repair group, *CM* Constant Murley Score, *ASES* American Shoulder and Elbow surgeons score, *VAS* visual analogue scale, *SD* standard deviation;

### Sensorimotor abilities

**Fig. 1:**
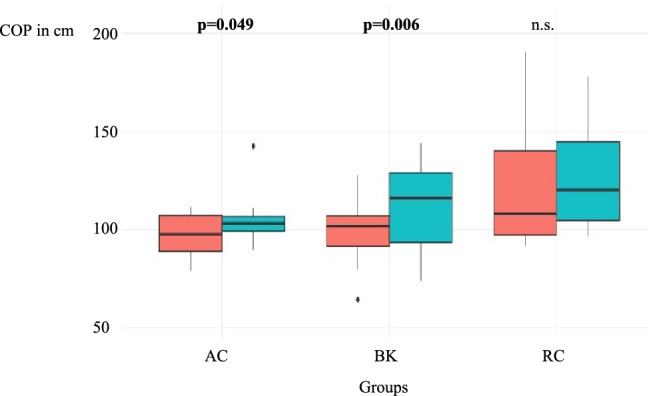
Boxplot: Comparison of intervention groups with treated side (blue) vs. contralateral side (red) with eyes open in cm

The results of the COP testing are demonstrated in Table 3. At final follow-up, there was a significant difference in COP length between the treated side and the healthy, contralateral side with eyes open for the ACJ-group (*p* = 0.049) and the BR-group (*p* = 0.006) (Fig. 1). However, there was no significant difference for the RC-group between the treated side and the healthy, contralateral side (n.s.) when assessed with eyes open or closed.

**Table 3:** Results of COP testing in cm for each group

	EO-SS			EC-SS			EO-NSS			EC-NSS		
	Med	25% quartile	75% quartile	Med	25% quartile	75% quartile	Med	25% quartile	75% quartile	Med	25% quartile	75% quartile
ACJ-group	103	99	107	92	89	115	98	89	107	92	85	106
RC-group	120	105	145	104	97	160	108	97	140	110	93	156
BR-group	116	93	129	106	89	121	102	92	107	106	90	116
Control-group	105	95	120	103	93	113	100	92	134	104	97	113

*EO-SS* Eyes open surgery side, *EC-SS* Eyes closed surgery side, *EO-NSS* Eyes open non-surgery side, *EC-NSS* Eyes closed non-surgery side, *Med* Median, *ACJ-group* acromioclavicular joint group, *RC-group* rotator cuff repair group, *BR-group* Bankart repair group

## Discussion

The most important finding of the study was that significant sensorimotor deficits persist in patients after successful surgical treatment at a mean follow-up of 32 months. More importantly, these sensorimotor deficits were noted in entirely asymptomatic patients with confirmed successful postoperative clinical and functional results, confirming the stated hypothesis. These findings highlight the importance of adequate training and rehabilitation protocols in patients after undergoing surgery of the upper limb. This may be of clinical relevance, as persistent sensorimotor deficits may subsequently reduce glenohumeral stability, thus potentially increasing the risk of re-injuries.

The clinical relevance of deficits in sensorimotor function despite good outcomes in clinical scores has already been indicated in the literature [17]. Beitzel et al. recently demonstrated that elite youth javelin throwers demonstrated structural changes, even though they did not present with a history of pain or injury of the shoulder, underlining the importance of continuous postoperative rehabilitation [18]. Clinically, this may be one of the major obstacles to return to full shoulder function after injury and surgical intervention.

It can be assumed that patient-reported outcome measures (“PROs”) alone may not be good enough to effectively measure the outcome of surgery of the upper extremity, especially in high-level athletes [19].

Furthermore, the data of this study demonstrated that the control group had overall low COP-results and therefore low stabilometric deviation, reproducing the results of Edouard et al. [2, 8]. Interestingly, the RC-group showed high deviations in COP measurement. This may be explained by the importance of the rotator cuff to contribute as an active stabilizer [4, 20, 21]. If this balance of forces is disturbed, glenohumeral joint kinematics change, which negatively impacts the sensorimotor function [7]. Another explanation may be the high age of the RC-group and the mostly traumatic history of ACJ injuries and glenohumeral instability that may suggest a presumably higher level of activity, which could have favorably influenced the execution of the measurement position. However, these results indicate that greater deficits in sensorimotor functionality occur after surgery, especially if the rotator cuff is involved, and may persist despite successful surgical intervention and postoperative rehabilitation.

Additionally, the injury pattern of ACJ separations does usually not cause lesions of the rotator cuff or capsule, as the most important structure for passive joint stability [20, 22, 23]. Interestingly, Witherspoon et al. demonstrated the existence of mechanoreceptors in the glenoid labrum and their importance for proprioceptive involvement in dynamic joint stabilization [24]. Clinically, Aboalata et al. showed that, despite good clinical outcomes, less than half of the patients achieved their previous level of exercise at a mean follow-up of 13 years after arthroscopic Bankart repair. This could indicate that, regardless of biomechanically sufficient restoration of the static joint stabilizers, sensorimotor deficits may persist [25].

The present study has several limitations. First, the study design does not include preoperative data regarding the sensorimotor ability of patients. However, the control group demonstrated that the contralateral side allowed for good comparability. Second, the subgroups significantly varied in their demographic composition. However, the purpose of this study was to include different types of shoulder injuries to investigate the sensorimotor impact for different structures of the shoulder joint. Third, the sample size of each subgroup is limited. However, the respective group selection was very homogeneous, which led to a very strict selection process to limit the effect of confounders. Fourth, the impact of the shoulder surgeries was compared to the contralateral, healthy side. Comparison to a healthy control group could be performed in a prospective setting to further sharpen the preliminary results. Finally, a standardized method to assess the sensorimotor function of the shoulder is yet to be determined. Other commonly used methods are the joint position sense and kinesthesia examinations [26–31].

Despite these limitations, the present data demonstrated that functional deficits even persist in entirely asymptomatic patients with confirmed successful clinical and functional outcomes after completing postoperative rehabilitation, indicating that rehabilitation protocols may require optimization. This is of clinical importance as it may lead to better clinical outcomes, higher patient satisfaction and performance, and reduced risk of re-injury in the long term. [32] This could be implemented into day-to-day clinical practice, if rehabilitation protocols include sensorimotor training components, balancing and strengthening components, and posture optimization. [33] Consequently, this allows the surgeon to discuss the importance of postoperative sensorimotor training with the patient, as specialized instrumentation and prolonged rehabilitation protocols are needed. With more severe impairment of static and dynamic shoulder stabilizers, a greater postoperative sensorimotor deficit can be assumed.

## Conclusion

Centre of pressure measurement detected sensorimotor functional deficits following surgical treatment of the shoulder joint in patients with confirmed successful clinical and functional outcomes. This may indicate that specific postoperative training and rehabilitation protocols should be established for patients who underwent surgery of the upper extremity. These results underline that sensorimotor training should be an important component of postoperative rehabilitation and physiotherapeutic activities to improve postoperative function and joint control.
